# Microglia in Epilepsy: From Molecular Mechanism to Therapeutic Strategy

**DOI:** 10.3390/cells15090835

**Published:** 2026-05-02

**Authors:** Yam Nath Paudel, Efthalia Angelopoulou, Sai Kulkarni, Robert E. Blair, Laxmikant S. Deshpande

**Affiliations:** 1 Department of Neurology, School of Medicine, Virginia Commonwealth University, Richmond, VA 23298, USA; robert.blair@vcuhealth.org; 2 1st Department of Neurology, Eginition Hospital, National and Kapodistrian University of Athens, 11528 Athens, Greece; angelthal@med.uoa.gr; 3 Department of Pharmacotherapy and Outcomes Sciences, School of Pharmacy, Virginia Commonwealth University, Richmond, VA 23298, USA; kulkarnis5@vcu.edu; 4 Department of Pharmacology and Toxicology, School of Medicine, Virginia Commonwealth University, Richmond, VA 23298, USA

**Keywords:** epilepsy, microglia, seizure, minocycline, neurodegeneration, neurogenesis, microglia depletion

## Abstract

**Highlights:**

1. Microglia, a resident immune cell of the CNS, have been widely acknowledged as a driver of neuroinflammatory cascades and possess a pathogenic and beneficial role in epileptic seizures.
2. In recent years, a large body of data has arisen regarding depleting (eliminating) microglia either via pharmacological or pharmacogenetic elimination.
3. Targeting microglial activation/modulating microglial phenotype against experimental seizure models has produced encouraging data.
4. Lack of robust and clinical data from microglia-targeted therapies warrants further understanding of epilepsy, investigating microglia in a real animal model of epilepsy.

**Abstract:**

The limit of disease-modifying therapeutic strategies against epilepsy has prompted mainstream epilepsy research toward understanding the pathways contributing to epileptic seizures. Microglia, the powerhouse of the brain’s innate immune system, is known for its role in epileptic seizures, contributing via neuroinflammation, neuronal death, and neurogenesis. Therapeutic targeting of microglia with its inhibitor and therapeutic compounds modulating its activation reduces the development of spontaneous recurrent seizure after status epilepticus in a pre-clinical model. Herein, we review various aspects of microglia in epilepsy, including their contribution to seizure-induced neuronal death and neurogenesis, the outcome of depleting microglia (both pharmacologically and genetically), the aspects of microglia–astrocyte interaction, and promising therapeutic outcomes achieved by targeting microglia.

## 1. Introduction

Epilepsy is a devastating central nervous system (CNS) disorder characterized by the repeated occurrence of seizures impacting over 70 million of the global population; however, the underlying mechanism of seizure generation has not been entirely understood [1,2]. Spontaneous recurrent seizures (SRSs), the significant hallmark of epilepsy, have been reported to be associated with increased neuronal excitability and hypersynchrony, and are mostly caused by brain trauma and genetic factors [3]. Plethora of CNS insults, including status epilepticus (SE), traumatic brain injury (TBI), and stroke, might be the leading cause of acquired epilepsy accounting for 20–30% of total epilepsy cases [4,5].

The development of epilepsy and spontaneous seizures is known as epileptogenesis [6], and its underlying mechanism is still elusive. There is an urgent need to develop novel therapies against epilepsy that can overcome the limitations of mainstream anti-seizure medications (ASMs), as they only provide symptomatic relief, leaving one-third of epileptic patients resistant to ASMs [7]. The plausible reason behind the therapeutic ineffectiveness of ASMs might be attributed to the fact that they do not target crucial mechanisms, including those involving non-neuronal cells [8,9]. Hence, as brain-resident immune cells of the CNS, microglia have attracted much attention in recent years in epilepsy research with potential to represent an emerging novel anti-epileptic therapy [10,11,12,13,14,15,16,17].

Microglia account for around 10% of the total adult CNS cells [18,19]. Microglia possess beneficial roles, including brain surveillance, neurogenesis, synaptic remodeling, neuronal excitability, vascularization, formation and maintenance of the blood–brain barrier (BBB), glial support, etc. [20,21,22,23]. During physiological conditions, microglia are mostly ramified cells with dynamic processes that safeguard their surrounding environment to monitor neural homeostasis [24]. On the contrary, during pathological conditions (including seizures), microglia turn to a reactive state, gain amoeboid shapes, and produce diverse inflammatory mediators such as cytokines, chemokines, and complement proteins [25].

Microgliosis is among the common process that contributes to epileptogenesis, as evident in an animal model of acquired epilepsy and drug-resistant epilepsy patients [26,27]. The role of microglia in epileptogenesis has been acknowledged since neuronal injury causes microglial activation and neuroinflammation, which likely contribute to epilepsy [28]. Interestingly, based on the stages of pathogenesis, microglia may have a differential role in epilepsy, and they exert both pro-epileptic and anti-epileptic effects [29]. For example, after temporal activation post-SE, microglia may inhibit the formation of aberrant neural circuits, neuronal death, and hyperactivation of neurons, exerting anti-epileptic effects [17,30]. On the contrary, the pro-epileptic effect of microglia is attributed to prompting neuronal hyperactivity and neuronal cell loss via inflammatory phenomena [31].

A deeper understanding of the role of microglia in seizure and epilepsy is needed, which is discussed in this review based on the recent advances and covers different aspects, including the role of microglia in seizure and its underlying mechanism, microglia dynamics in seizure, pre-clinical evidence targeting microglia in epilepsy, and its future translational implications.

## 2. Literature Review Strategy

The literature search was carried out on the PubMed database and Google Scholar using the following keywords: “Epilepsy”, “Microglia”, “Seizure”, “Minocycline”, “Neurodegeneration”, “Neurogenesis”, “Microglia depletion”. A combination of these keywords was used to gather high-quality relevant publications ranging from 1992 to 2026, mainly selected based on their relevance to the topic. However, most references were published between 2010 and 2026, accounting for more than 75% of the total references.

## 3. Microglia and Epilepsy

A mounting amount of evidence supports the role of neuroinflammation in the pathogenesis of epilepsy [31,32,33]. Microglia are highly reactive during SE [17], which are characterized by an ameboid morphology [34,35], increased production of pro-inflammatory cytokines [36,37,38], proliferation, and increased K^+^ conductance [39]. A compelling link between microglia and epilepsy has been implicated in the past decades of research [17,40]; however, the underlying mechanism of microglia contribution to the pathogenesis of epilepsy is yet to be fully understood.

### 3.1. Microglial Responses to Epileptic Seizures

It is largely understood that microglial activation occurs post-seizure induction in a pre-clinical model of epileptic seizures. Microglial activation is characterized by changes in cell morphology and biochemistry [41]. The size and shape of the cell body, the number, length and orientation of cellular processes, and the number and length of the branches are among the criteria that characterize microglial modifications during their activation [42]. Hippocampal microglia become activated after SE induction, even before the onset of neurodegeneration [43].

Intraperitoneal (I.P.) Kainic acid (KA)-induced SE leads to an acute (24 h) and sustained (7 days) activation of microglia as evident by an upregulation in the area of fluorescent cells in the hippocampal region (hilus), microglial cell numbers, and microglial aggregates in the CA3 region of KA-treated mice when compared to normal controls [44]. Significant upregulation in IBA1 immunoreactivity in the hippocampal CA3 region was observed on day 3 and day 7 after intracerebroventricular (ICV) KA administration, and Western blot evaluation (12 h., 1 d, 2 d, and 3 d; hippocampal tissues) showed that starting at 1 day, the IBA1 content was two-fold more significant than in artificial colony-stimulating factor (aCSF)-injected control mice [13]. The degree of seizure severity is correlated with microgliosis and microglia activation, as evident by increased upregulation of IBA1-positive cells (peaked at 3 d and returned to normal at 14 days) following SE [16]. In the I.P. KA-induced SE model, no microglial activation was observed in PN9 rats 4 h and 24 h after seizure onset, whereas strong microglial immunoreactivity (OX-42) was detected in PN21 rats, reflecting activated microglia accompanied by round to oval shape and developed process [45]. In an animal model of epileptogenesis induced by intrahippocampal KA (IHKA) injection, IBA1-positive cell activation was prevalent for 24 h and 3 months, and was accompanied by round to oval-shaped, hypertrophic processes with a reduction in distal ramifications [10]. An electrical-induced partial SE model in rats led to significant hippocampal microglial activation, and was accompanied by increased microglial cell numbers and the presence of activated microglial phenotypes (1 week after SE). Upon morphological analysis, ramified/surveying, intermediate, and round/ameboid morphologies were observed, reflecting an activated phenotype [46].

In a Pilocarpine-induced TLE model, hypertrophied IBA1-labeled microglial cells were observed in the hippocampal region (CA1 and CA3) and dentate gyrus (DG) (1 day after TLE) [47]. Microglia at this time point were activated as evidenced by pleomorphic, thickened processes and increased branch points. Further morphometric analysis revealed significant differences in the mean area of IBA1-labeled processes and IBA1-labeled processes after seizures. In addition, the densiometric analysis revealed increased IBA1 immunoreactivity in hilus, CA1, and CA3 regions (1 day after seizure) [47]. The density of microglial activation in the hippocampus increased post-birth and reaches peaks at the second week of life, which is the age when animals are acknowledged to be more susceptible to seizure triggers (LPS-induced Febrile seizures and KA-induced SE). Interestingly, these animals showed increased microglia activation in the hippocampus after seizures compared to the other ages [48]. An increased number of OX-42-positive microglial cells (amoeboid morphology) reflecting microglial activation has been observed after 12, 24, and 48 h after Pilocarpine-induced SE [49].

Rapid and persistent activation of microglia/macrophages in the hippocampal CA1 region following KA-induced SE was evident based on morphological analysis of density, cell volume, ramification index, and process length of IBA1-positive cells [50]. In a recent study with an in vitro KA-induced seizure model, quantitative and morphological evaluation of microglia in the CA3 region suggests significant morphological alterations of microglia in the KA group, accompanied by a thicker cytoplasmic projection of microglia, and larger and amoeboid cell bodes, reflecting a reactive/activated microglia [51]. However, electro-convulsive seizures (ECSs) do not change the morphology or activation state of resting microglia as evidenced by no effect of ECS on branch length, the number of endpoints, or the calculated fractal dimension during morphological analysis [52]. It is worth mentioning that all these microglia changes after SE induced with different experimental models are descriptively lacking the precise dynamics of microglia activation in specific brain regions. A better understanding of microglial activation patterns will indeed expand our knowledge of the short- and long-term consequences of SE [53].

The very first study investigating real-time microglial dynamics after severe seizures (ICV-KA) reported the formation of microglial process pouches (MPPs) that target but rarely engulf beaded neuronal dendrites [12]. These MPPs increase their motility/chemoattractant properties and do not possess phagocytic activity; most MPPs (70%) are maintained rather than engulfed, and formation and maintenance of MPPs are independent of chemotactic (CX3XR1 and P2Y12) and phagocytic (TREM2 and P2Y6R) receptors [12]. In a similar line of study by the same research group, daily rearrangement of the microglial landscape has been documented in young adults using in vivo two-photon imaging, and this microglial landscape can be modulated by severe seizures. Precisely, the microglial rearrangement was increased within the first 24 h of KA-induced seizures, whereas after the third day of KA-induced seizures, the basal levels were restored and maintained for a month [54]. A similar increase in microglial rearrangement was observed within 24 h of Pilocarpine-induced seizures. These findings reflect that the global increases in neuronal activity due to seizures lead to increased microglial somatic rearrangements [54].

### 3.2. Microglial Proliferation in Seizures

Microglial proliferation is a natural response to insult and a crucial prerequisite for microglia to execute their functions [55]. However, inappropriate microglial proliferation (too much or too little) may result in negative CNS health [55]. Timely manipulation of microglial proliferation, particularly via CSF-1R signaling, might represent a potential target for improving epilepsy outcomes [10].

The differential role of microglial proliferation in distinct phases of diseases, i.e., in the early disease phase and established chronic epilepsy, has been reported, where blockade of microglial proliferation with GW2580 during intra-amygdala KA-induced SE and early thereafter neither impacts SE nor halts epilepsy development [10]. However, GW2580 administration during the chronic phase of epilepsy contributes to seizures as evidenced by a reduction in spontaneous seizures [10]. Blockade of microglial proliferation with GW2580 is associated with reduced hippocampal leukocyte infiltration observed within 1 week of SE. Microglia proliferation during the early post-injury period does not contribute to neuronal network hyperexcitability underlying seizure generation because GW2580-treated naive mice did not exert any changes in the basal excitatory neurotransmission and neuronal excitability [10]. Nevertheless, the factors driving microglial proliferation during seizures are not yet fully understood.

Only resident microglia, but not monocytes, proliferate in the hippocampus after SE. In an ICV KA-induced SE model, significant Ki67 and BrdU staining co-localized with IBA1-positive cells were observed in the CA1 and CA3 hippocampal region after KA-induced seizures (1 d, 2 d, 3 d, and 7 d) [16]. Moreover, only GFP^+^ cells were positive for Ki67 in CX3CR1^GFP/+^: CCR2^RFP/+^ mice and it was found that around 59.2% and 45.1% of microglia were Ki67^+^: GFP^+^ in CA1 and CA3 (3 d after KA), respectively. In addition, Ki67 localization was only in observed in CD11b^+^: Tdtomato^+^ resident microglia, but not in CD11b^+^: Tdtomato–monocytes, and no co-labeling of CD169 (monocyte marker) with Ki67 was observed. This finding suggests that only resident microglia, but not infiltrating monocytes, proliferate after seizures in the hippocampus, and proliferation of resident microglia is the major source of microgliosis after SE [16].

In the quest for understanding an underlying mechanism of microglial proliferation and its correlation with KA-induced SE, it was found that microglial proliferation is CSF-1R-dependent, as evidenced by a significant reduction in microglial proliferation (Ki67 staining) (3 d after SE) after treatment with CSF-1 antibody and GW2580 [16]. GW2580 treatment affected microglial morphology, accompanied by reduced primary microglial branch numbers, microglial soma size, and reduced monocyte (CD169^+^ cells) infiltration compared with untreated mice after KA treatment. However, no microglia apoptosis with cleaved caspase-3 staining was observed post-CSF-1Ab treatment, and it was confirmed that GW2580 and CSF-1Ab reduce microglial proliferation via BrdU labeling [16]. However, an earlier study has reported the crucial role of toll-like receptor 2 (TLR2) in KA-induced microglial proliferation as evident by the reduction in Iba1-IR observed in TLR2 KO mice compared with WT mice, and this difference was mainly due to differences in microglia proliferation and activation, but not differences in macrophage infiltration [56].

It is worth noting here that inhibiting microglial proliferation (with GW2580 and a CSF-1 antibody) reduced neuronal death induced by KA, as evidenced by reduced FJC-positive cells, but it did not alter seizure behavior [16].

### 3.3. Microglia and Neurodegeneration in Seizures

Neurodegeneration is the most widely discussed topic in relation to epilepsy and has been widely acknowledged in the disease pathogenesis [57,58,59]. In an experimental seizure model, neurodegeneration has been associated with innate immune activation and upregulated expression of pro-inflammatory cytokines [60,61]. Unarguably, direct and indirect interactions between neuronal damage and cell death have been prevalent in animal models of SE and in human patients [62,63,64,65,66], and delineating the mechanism of SE-induced neuronal death might help to suppress the deleterious effects of SE [67]. The widely accepted mechanism of neuronal cell death in SE includes apoptosis, necroptosis, pyroptosis, ferroptosis, and autophagy [67].

There is an increased understanding that microglia exert a crucial role in seizure-induced neurodegeneration mainly via the CX3CR1 receptor, which is evident by the reduction in seizure-induced neurodegeneration (reduced FJC^+^ cells in the hippocampus 1 week after SE) upon treatment with CX3CR1 antibody [46].

The complement pathway (C3-C3aR) has been reported to mediate seizure-induced neurodegeneration in the ICV KA-induced SE model. Reduced FJC^+^ neurons (in the hippocampal CA3 region) were observed in C3^−/−^ mice but not in C3aR^−/−^ mice when compared to the WT mice [13], reflecting that C3^−/−^ can rescue against KA-induced neurodegeneration. The disparities in the findings between KA-induced neurodegeneration in C3^−/−^ mice and C3aR^−/−^ might be due to the fact that C3 cleavage generates C3b, correlating with apoptotic neurons via CR3 [68], and ultimately, C3b production might result in cleavage of C5, which can contribute to neuroinflammation in epilepsy [69,70].

In an experimental model of Pilocarpine-induced MTLE, microglial activation only partially correlates with segmental neurodegeneration, as most of the neuronal death in the hilus, CA3, and CA1 regions correlates with an increased number of CD11b. In addition, microglial activation is observed in the region, including molecular and granule layers of DG that do not feature neurodegeneration [71].

Microglial function in neurodegeneration and seizures has been mostly validated using pharmacological approaches, such as minocycline and microglia deletion (both pharmacological and genetic). Minocycline, an inhibitor of microglial activation, reduced SE-induced neuronal loss and reduced production of pro-inflammatory cytokines IL-1β and TNF-α (in the hippocampal CA1 and the adjacent cortex), without affecting astrocytic activation [72]. Inhibition of TNF-α levels has been associated with reduced epileptic activity [73], and IL-1β plays a role in neuronal excitability by increasing glutamate concentration [74], potentiating the function of the neuronal NMDA receptor [75], etc. The disease-modifying effects (reduction in frequency, duration, and severity of SRS) observed with Minocycline treatment may be partially due to its inhibitory effect on SE-induced IL-1β and TNF-α production [72]. Moreover, Minocycline exerted neuroprotective effects as evident by increased NeuN^+^ cells in the Minocycline-treated group compared to the SE group (14 days post-SE), and these neuroprotective effects may be in part mediated by the ability of Minocycline to suppress the SE-induced activation of microglia and the related pro-inflammatory cytokine production [72].

Minocycline, via inhibiting both caspase-dependent and -independent apoptotic pathways, contribute to the reduction in hippocampal damage after KA [76]. Treatment with Minocycline reduced DNA fragmentation, reflecting apoptosis and hippocampal cell damage after KA treatment. Further, Minocycline inhibited both caspase-dependent pathways, i.e., release of cytochrome c and cleavage of caspase-3, and the caspase-independent pathway, i.e., translocation of apoptosis-inducing factor (AIF) and large-scale DNA fragmentation post KA-induced seizure [76].

Neuronal damage was observed in the ICV KA-induced SE as evidenced by significant staining of FJC in the CA3 and CA1 regions during severe seizures, and microgliosis was reported as neuronal loss was accompanied by an increased number of IBA1^+^ cells. CSF-1 antibody and GW2580 treatment significantly reduced KA-induced neuronal death [16]. Genetic depletion of microglia worsened KA-induced neuronal loss as evidenced by increased FJC^+^ neurons in the hippocampal region of iDTA, iDTR, and CSF1R mice [14]. Microglia protect neurons from excitotoxicity-induced degeneration, which is evident by the fact that microglial depletion by CSF-1R inhibitor (PLX3397) aggravates excitotoxicity-induced neuronal degeneration after Pilocarpine-induced SE, as evident by increased FJC^+^ cells in the DG of PLX3397-treated group compared to the vehicle-treated group [11].

What can be conferred here is that, despite the method of microglia depletion, microglia are beneficial for maintaining neuronal survival and are naturally available to quench acute seizures and further protect neurons against excitotoxicity-induced degeneration [11]. However, it would be indeed interesting to know more about how microglia can immediately respond to neuronal overexcitation and take a protective role against seizures and neuronal excitotoxicity, and what might be the underlying mechanism [11]. 

### 3.4. Interaction Between Microglia and Infiltration of Peripheral Immune Cells

Circulating monocytes penetrate the damaged or diseased brain in response to the injury instead of penetrating the healthy CNS and further contribute to an immune response [38]. Epileptic seizures lead to activation of the neuroinflammatory response, disruption of the BBB, upregulation of chemokine production (CCL2), infiltration of monocytes into the CNS, and activation of brain-resident microglia and reactive astrogliosis. Infiltrating monocytes, by interacting with their microglial counterpart, drive microgliosis and neuronal cell death in a pre-clinical SE model [16,77].

A better understanding of the fate of peripheral monocytes infiltrating the brain following SE is very crucial in developing therapies, as they might be a promising cellular target ameliorating SE-related neurodegeneration and neurobehavioral comorbidities. The KA and Pilocarpine-induced SE model triggers blood-borne C-C chemokine receptor type 2 positive (CCR2^+^) monocyte recruitment into the brain [38,78]. On the contrary, blocking the recruitment of monocytes using global CCR2 ko or systemic CCR2 antagonist (INCB3344) attenuates SE-induced significant neuronal loss, alleviates long-term recognition memory deficits, and reduces SE-induced deficits in working memory [38,78].

In an experimental model of IHKA-induced new-onset refractory status epilepticus (NORSE), robust hippocampal microglial and immune activation has been observed. Using Cx3Cr1 ^GFP/+^ Ccr2 ^RFP/+^ reporter mice that separate resident microglia from infiltrating monocytes, simultaneous activation of resident microglia (GFP^+^) and infiltration of peripheral monocytes (RFP^+^) has been observed in the hilus at day 7 post-IHKA [79]. Furthermore, profound upregulation of RFP^+^ monocytes in the hippocampi (hilus) of IHKA-injected mice compared to sham mice indicates plausible BBB disruption and recruitment of peripheral immune cells [79].

Infiltrating monocytes exert their anti-inflammatory phenotype during an early phase of epileptogenesis or via reactivating it during the course of epilepsy, reflecting it as a promising therapeutic avenue [80]. Infiltrating monocytes penetrate the brain 24 h post-SE, where microglia plausibly regulate an early pro-inflammatory response, as evidenced by a 14-fold upregulated TNFα transcript level in microglia when compared to monocytes [80]. However, at 24 h post-SE, infiltrating monocytes display an anti-inflammatory and neuroprotective phenotype.

Plasma sample analysis from drug-resistant epilepsy (DRE) patients exhibited an upregulated percentage of monocytes expressing activation marker CD11b, when compared to psychogenic non-epileptic seizures (PNESs). In addition, an increased number of “classical” monocytes (CD14++CD16−) and reduced levels of “non-classical” monocyte (CD14−CD16+) subsets were observed in the DRE group [81]. The DRE monocytes express CD11b and P2X7R, reflecting an activated state, suggesting that pro-inflammatory peripheral immune alterations occur in DRE patients compared with the PNES controls [81].

Taken together, preventing CNS infiltration of monocytes is neuroprotective, resulting in ameliorating BBB damage, suppressing neurodegeneration, ameliorating neurobehavioral comorbidities, etc., representing a promising translational opportunity for developing therapies.

### 3.5. Microglia, Neurogenesis, and Seizure

Microglia contribute to regulating each stage of adult neurogenesis (proliferation, survival, and maturation) in both homeostasis and epileptic conditions [82]; however, the underlying mechanism behind the role in the integration of new cells is yet to be fully understood. Microglia contribute to the epileptogenic process plausibly via neurogenesis and modulation of the neuronal network [83].

An animal model of SE enhances adult neurogenesis, resulting in an increased number of newborn granule cells [84,85], and microglia engulf viable neurons after SE in the hippocampus [86]. After SE, microglia regulate neurogenesis, mainly by engulfing excess newborn cells in the DG. In addition, inhibiting microglial activation using Minocycline promoted the survival of the newborn cells [30].

In the adult hippocampal subventricular zone (SVZ), non-inflammatory microglia maintain homeostasis by removing excess newborn cells via apoptosis-coupled phagocytosis [87]. However, after KA-induced TLE, microglia could not remove newborn apoptotic cells, leading to their accumulation in the DG region [86], where phagocytic impairment is paralleled by reduced motility, decreased levels of Trem2, MerTK and CR3, and upregulated expression of inflammatory cytokines [86]. Despite decreased phagocytosis of apoptotic cells, microglial engulfment of viable non-apoptotic cells was reported in the DG during epileptogenesis [30,86]. Apoptotic and viable newborn cells in the DG are phagocytosed and eliminated by microglia post-SE, indicating the crucial role of microglia in neurogenesis and in maintaining homeostasis of the dentate circuitry after epileptic seizures [30].

In the KA-induced SE model, upregulation of BrdU-retaining and doublecortin (DCX) (marker of immature neuron)-expressing new and immature neurons was detected in the DG (both the subgranular zone and the hilus) of TLR9-KO mice, indicating that loss of TLR9 promotes KA-induced aberrant neurogenesis [88]. Minocycline treatment leads to an exacerbation of seizure-induced aberrant neurogenesis via TLR9 signaling, reaching the BrdU and DCX levels similar to those of the TLR9-KO mice treated with KA alone [88]. On the contrary, TNF-α derived from microglia alleviates seizure-induced aberrant neurogenesis [88]. This might be because microglia used their receptor TLR9 to sense self-DNA, which might be released from the dead neurons, and then secreted TNF-α, suppressing the proliferation of neural stem cells [88].

Minocycline treatment in the Pilocarpine-induced SE model reduced aberrant migration of newborn neurons at 14 days after SE and further reduced the ectopic hilar processes of newborn neurons, preventing them from extending deeply into the dentate hilus [89]. In the ICV KA-induced seizure model, increased expression of DCX-positive cells, Ki67-positive cells, and DCX ^+^: Ki67^+^ double-positive cells were observed at 3 d and peaking at 7 d after ICV KA. A similar line of increment was observed with DCX^+^: BrdU^+^-positive cells 7 d after SE. All these suggest a KA-induced increase in DG neurogenesis and report that KA-induced SE significantly increases the projections of DCX-immature neurons [15].

Furthermore, to explore the role of microglia in neurogenesis and immature neuronal projections after SE, a genetic microglial ablation approach was used. The results showed that DCX- and Ki67-positive cells were significantly reduced compared to the non-ablated group, suggesting a role for microglia in promoting ongoing neurogenesis in the steady state. Microglia promote both basal and seizure-induced neurogenesis in the adult DG and steady-state and seizure-induced projection of immature neurons after SE [15]. in terms of molecular mechanisms, it seems that microglial P2Y12 plays a role in promoting seizure-induced neurogenesis and immature neuronal projections [15]. However, it is still unknow how microglial P2Y12 activation triggers neurogenesis. Future studies are needed to address which microglial factors are involved in neurogenesis. Microglial activation may be an effective way to remove ectopic newborn cells, providing aberrant excitatory networks in the DG post-SE, which in turn may prevent the resultant epileptogenic processes [82].

### 3.6. Microglia–Astrocytes Communications in Epilepsy

Microglia–astrocyte communication resembles a delicate balance that regulates neuronal cell functions during health and disease and maintains homeostasis during physiological conditions [90].

Hypertrophied GFAP-labeled astrocytes have been observed in the hippocampal CA1 and CA3 regions and DG after 1 day of Pilocarpine-induced TLE [47]. Similarly, astrocytes in the mouse dentate hilus degenerate in an animal model of Pilocarpine-induced SE [91]. In an IHKA-induced MTLE model, significant astrocyte immunoreactivity was observed in the ipsilateral hippocampus region and increased astrocyte activation during epileptogenesis, which was further sustained until the chronic phase of the diseases [92].

In an experimental model of Pilocarpine-induced SE, microglia and astrocyte activation both contribute to epileptogenesis collaboratively. Astrocyte activation was followed by microglial activation, where the area of IBA1^+^ microglia (1–7 days after SE) and GFAP^+^ astrocytes (3–28 days after SE) was increased in the CA1 region [93]. Furthermore, reactive astrocytes exhibiting greater IP3R2-mediated Ca^2+^ signals emerged after microglial activation (after SE), and genetic deletion of IP3R2 ameliorated both the aberrant Ca^2+^ signals in astrocytes and the increased seizure susceptibility. Pharmacological inhibition of microglial activation with Minocycline at the early phase after SE suppressed astrogliosis, accompanied by aberrant Ca^2+^ signals in astrocytes, and increased seizure susceptibility [93].

Interaction between microglia and astrocytes has been observed in the ICV KA-induced SE model, where IBA1 and GFAP immunoreactivity have been extensively increased at 3 d and 7 d after SE when compared to normal controls [13]. Confocal imaging suggests close spatial overlap between IBA1 and GFAP immunoreactivities in the KA group, providing compelling evidence of a physical microglia–astrocyte interaction after SE. More interestingly, microglial ablation with PLX3397 also reduced astrocyte activation as evidenced by fewer GFAP-positive cells in the PLX3397 group [13].

Microglia–astrocyte communication has been implicated in a plethora of neuroinflammation-mediated pathologies [94,95]. The bidirectional interaction between microglia and astrocytes is driven via their molecular secretions, which include TNF-α, Nitric oxide, Type 1 IFN-A, NADPH oxidase-derived H_2_O_2_, TGF-α, C1q, MCP-1, TGF-β, C3, CCL2, GDNF, etc. [96]. An ample amount of studies report microglia and astrocyte activation in an animal model of seizure; however, the precise mechanism regulating microglia–astrocyte crosstalk during a seizure is not yet fully understood. However, early upregulated C1q plays a crucial role in initiating microglia–astrocyte communication, which in turn drives synaptic disruptions, neuronal loss, and formation of disrupted neuronal networks [97]. Cytokines (IL-1β, TNF-α, and IL-6) have been acknowledged as a crucial mediator driving microglia and astrocyte communication [98]. Voltage-gated microglial receptor (HV1) and astrocytic IFN-IFN-γ mediates microglia–astrocyte interaction [99]. SE leads to microglial activation, leading to secondary astrocyte activation, which in turn secretes C3, activating microglia through C3aR signaling. Complement C3-C3aR signaling has been reported to regulate microglia–astrocyte interaction in KA-induced SE as evident by lower IBA1 and GFAP-positive cells and less co-localization of IBA1 and GFAP staining in C3^−/−^ and C3aR^−/−^ mice in comparison to WT mice [13].

The interlinkage among the mitochondrion–immunity–metabolism signaling axis mediates neuronal mitochondrial damage to chronic epileptic seizures [100]. Z-mitochondrial DNA (mtDNA)-facilitated activation of cyclic GMP-AMP synthase (cGAS)-STING and D-serine are crucial drivers of epilepsy initiation, unraveling insights into mechanistic neuron–microglia–astrocyte communication [100]. Single-nucleus RNA sequencing analysis from human brain tissues of TLE patients reveals that the *SPP1-CD44* axis has emerged as a driver mediating microglia and astrocytes signaling, where reactive microglia and reactive astrocytes act as primary SPP1 senders and CD44 receivers [101]. Though limited, these pathways driving microglia–astrocyte crosstalk not only unravel pathological microglia-mediated astrocyte activation and its consequences, but also provide an opportunity for future potential treatment avenues.

### 3.7. Microglial Ca^2+^ Signaling and Neuronal Activity in Epilepsy

Microglia contribute to the regulation of neuronal activity directly via communicating with neurons, or indirectly, through astrocytes, manipulating neurotransmission and synaptic function [102,103]. Microglia have an ability to sense neuronal activity changes under both hypoactivity and hyperactivity and much has already been reviewed [104]. A recent study has unraveled the distinct microglial dynamics driving neuronal network activity under awake and anesthetized conditions and reported that microglia in awake mice have reduced surveillance territory with decreased process area and motility, and reduced responsiveness to an acute injury compared to those in anesthetized conditions (with isoflurane or a fentanyl cocktail) [105,106].

Microglial Ca^2+^ signaling might plausibly contribute to microglia activation after CNS injury [107]. Recent findings have advanced this understanding by reporting that microglial Ca^2+^ signaling responds to the changes in neuronal hyperactivity in seizures [108]. Neuronal hyperactivity induced by KA increased excitatory Ca^2+^ activity immediately after administration (15–30 min) and after the first observed seizure, which was accompanied by increased microglial process Ca^2+^ activity. In case of KA-induced SE, altered morphology, as evident by increased process ramification, was exhibited by layer I microglia. Increased microglial Ca^2+^ was observed in microglial somata and processes, where Ca^2+^ signaling lasts up to 10 days. All these reflect the dynamic nature of microglial Ca^2+^ activity that can be changed after excitotoxicity [108]. Microglial process extension is temporally correlated and strongly associated with their Ca^2+^ activity. Furthermore, with a designer receptor exclusively activated by designer drugs (DREADD) approach using Gq DREADD activation in CaMKIIa to increase neuronal activity, DREADD ligand, Clozapine N-oxide (CNO) administration leads to greater microglial process Ca^2+^ activity and extension [108]. Findings form this study provide compelling evidence that microglial Ca^2+^ signaling might be implicated in excitotoxicity and other pathological conditions, including epilepsy [108,109].

ATP/ADP and purinergic receptors have been acknowledged as the agonist and transducers of microglia Ca^2+^ signaling [107,110,111], and Kainate and Gq DREDD system might plausibly increase the release of purinergic molecules [112], increasing both process growth [17,113] and process Ca^2+^ activity [114]. Though how microglia sense and regulate neuronal hyperactivity has been well discussed before [104], what is not well understood is the receptor mechanism mediating microglial Ca^2+^ signaling in seizures.

## 4. Beneficial Role of Microglia in Epilepsy

### 4.1. P2Y12 and CX3CL1/CX3CR1 Signaling Axis

The very pioneering study led by Eyo and the team reported the neuroprotective effect of microglial P2Y12 in an acute epilepsy model. The purinergic receptor P2Y12 is expressed in microglia but not in macrophages [115], and P2Y12l loss lead to the morphological transformation of microglia from a highly ramified to an amoeboid state [113]. Microglial P2Y12R detects synaptic release of ATP following neuronal activity and regulates chemotaxis and microglial motility, which are acknowledged to regulate microglia-mediated suppression of neuronal activity [116,117,118,119]. P2Y12 receptors may plausibly mediate microglial landscape rearrangement mainly via translocation mechanisms, and P2Y12 receptors are crucial for housekeeping surveillance of microglia [54].

Microglial process numbers were increased after acute seizure induced by ICV and I.P. KA administration, as evidenced by increased numbers of primary processes originating from microglial soma and increased microglial cell area [17]. Interestingly, acute seizure induction in P2Y12 KO mice resulted in decreased seizure-induced increases in the microglial process numbers and exacerbated KA-induced seizure phenotypes [17]. These neuroprotective effects of microglial P2Y12 in acute seizures might be because hyperactive neurons release ATP, which leads to microglia–neuron interactions mediated by P2Y12R during the acute seizure and, in turn, reduces neuronal activity via A1 receptors [118,120]. Moreover, an earlier study has unraveled the mechanism of microglia–neuronal interaction in acute epilepsy via modulation of microglial morphology in an NMDA-dependent manner [17]. The same research group has termed microglial process convergence (MPC) a novel form of microglial–neuronal interaction. Using in vivo 2-photon microscopy, they observed that a reduction in extracellular Ca^2+^ causes microglial processes to converge at distinct sites, termed MPC, and this MPC targets neuronal dendrites that are not dependent on neuronal action potential firing and are mediated by ATP release and microglial receptors (P2Y12) [121]. Global P2Y12 (gKO) and microglial-specific P2Y12 deficiency exacerbates a chemoconvulsive (KA)-induced seizure model, as seizure persisted in gKO mice compared to the WT; increased seizure score was also observed in P2RY12 cKO mice compared to littermate P2RY12 cWT mice [122]. P2RY12 signaling maintains microglial structural complexity in epileptic seizure, as evidenced by a substantial reduction in process branching in gKO microglia in comparison to WT microglia. P2RY12 deficiency increased neuronal activation, demonstrating exaggerated cFos response in P2RY12 KO mice, reflecting that microglial P2RY12 activity is required to limit excessive neuronal firing during KA-induced hyperexcitability. P2RY12 signaling regulates inhibitory synaptic coverage (in the absence and presence of seizure activity) as evidenced by the significant reduction in VGAT-positive area in gKO mice [122]. CX3CL1 (Fractalkine) is a transmembrane chemokine that is expressed by both neurons and glia, whereas its receptor, CX3CR1, is mainly expressed in microglia [46]. The reciprocal interaction between the microglial chemokine receptor and the neuronal ligand CX3CL1 leads to microglia–neuron communication that plausibly regulates an array of brain functions including neuronal networks, synapse maturation and plasticity, regulation of the immune process, and cognitive function [123,124,125]. CX3CL1 acts on the G protein-coupled receptors (CX3CR1) expressed on microglial cells, and in turn regulates their activation, migration, and phagocytic activity [126,127]. The CX3CL1/CX3CR1 signaling axis exerts a crucial role in SE-induced neuronal damage via neuron–microglia interactions [128]. In the Pilocarpine-induced model of SE, increased CX3CR1 immunoreactivity was observed in the hippocampal neurons (between 1 h and 1 day) post-SE. CX3CR1 neutralization, in turn, decreased the number of FJB^+^ neurons and alleviated microglial activation post-SE, reflecting that neuronal CX3CR1 expression may contribute to neurodegeneration after SE through the promotion of microglial function [128]. In the electrically induced partial SE model, increased hippocampal microglial activation was accompanied by increased microglial cell numbers, and activated microglial phenotypes were observed one week after SE. In the SE group, a reduced percentage of surveying/ramified microglia (by about 85%) increased intermediate and round morphology, reflecting that an activated phenotype was observed in the GCL, molecular layer, and dentate hilus. Hippocampal cell death occurred in the granule cell layer and the dentate hilus of the SE group, as evidenced by the increased numbers of FJC^+^ cells. Moreover, the non-significant difference in expression of CX3CL1 was observed in the GCL and dentate hilus of the SE group, whereas significantly increased CX3CR1 expression was observed in the DG of SE rats. CX3CL1 infusion after SE did not affect IBA1^+^ cell counts, the percentage of IBA1^+^ microglial cells expressing ED1, and several proliferating Ki67^+^ cells. On the contrary, infusion with a lower dose of CX3CR1 antibody one week after SE reduced IBA1^+^ cell counts, the percentage of IBA1^+^ microglial cells expressing ED1, and the number of proliferating Ki67^+^ cells in the granule cell layer, molecular layer, and dentate hilus. These findings strongly reflect the role of the CX3CL1/CX3CR1 signaling axis in limbic seizure-induced hippocampal pathologies ranging from microglial activation to neurodegeneration [46]. CX3CL1 signaling has been shown to regulate physical microglial–neuronal interactions or MPC post-SE. The experimental seizure model (induced by I.P. KA and Pilocarpine) triggered MPCs, and these MPC events were independent of the ionotropic glutamate receptor function and firing of an action potential. However, these MPCs were significantly reduced when NMDA receptors were antagonized, reflecting the need for NMDA receptors for the induction of seizure-induced MPC events [129]. CX3CL1 signaling is sufficient for the initiation of MPC and is required for glutamate- and seizure-induced MPCs, suggesting that molecular regulation of MPC depends on CX3CL1 signaling via IL-1β release. Moreover, CX3CR1 deficiency led to increased seizure phenotypes and animal mortality, correlating with decreased MPCs, which reflects the neuroprotective potential of MPCs [129].

All the studies discussed above demonstrate the neuroprotective nature of microglia as evidenced by the fact that genetic deletion of P2Y12 exacerbates acute seizures and genetic depletion of microglia worsens SE and chronic seizures [14,128,130]. The mechanism underlying microglial neuroprotection during seizure onset is mediated by microglial Gi-Dreadd (expressing Gi-Dreadd in microglia with CX3CR1 as inducible promoter) as evidenced by reduced seizure severity in the ICV KA model of SE [131].

### 4.2. Microglia Depletion (Pharmacogenetic and Pharmacological Elimination)

Microglia depletion is an effective and emerging strategy for studying the role of microglia in health and disease, as well as microglial repopulation in the CNS [132,133]. In the context of epilepsy, several pharmacological and genetic strategies have been used to deplete microglia [10,14]. Microglial survival, maintenance, and proliferation mainly depend on the colony-stimulating factor 1-receptor (CSF-1R) [134,135], and CSF-1R inhibitors have been widely used to deplete microglia in the CNS [136]. The extensively used CSF-1R inhibitors include PLX-3397, PLX-5622, and GW2580 [10,14,132].

Multiple genetic strategies of microglia depletion using iDTA mice (expressing diphtheria toxin A in CX3CR1^+^ cells in CX3CR1^CreER/+^: R26^iDTA/+^ mice), CSF-1R mice (CSF-1R knocked out using CX3CR1^CreER/+^: Csf1r^Flox/Flox^ mice), and iDTR mice (CX3CR1^CreER/+^: R26^iDTR/^) showed that microglia possess a protective role both in acute and chronic seizure models [14]. In an acute seizure model induced via ICV administration of KA, microglia depletion aggravates seizure severity and increased mortality in all iDTA, CSF-1R, and iDTR mice. In addition, significant neuronal loss was observed as evidenced by the FJC-positive neurons in the hippocampal region (CA1 and CA3) of iDTA, CSF-1R, and iDTR mice. Similarly, microglia depletion has promoted KA-induced SRS in chronic epilepsy models induced with IHKA injection. It is worth mentioning that, microglia, when repopulated upon ceasing Tamoxifen injection, reversed the detrimental effect of microglia depletion on seizure, as evidenced by no significant difference in seizure score among iDTR and CSF-1R mice and their respective controls [14]. This reflects the beneficial effect of microglia on regulating seizure severity induced by KA. Similar lines of findings have been reported for microglia depletion via PLX3397 treatment in a Pilocarpine and PTZ-induced model of seizure [11]. Seizure severity was worsened in the PLX3397-treated group in the Pilocarpine model, whereas the number of mice reaching generalized seizures and the number of EEG spike discharges was higher in the PTZ model [11].

PLX3397 administration has provided an anti-epileptic effect in an array of pre-clinical epilepsy models [137]. Administration of PLX3397 (3 or 30 mg/kg per day; for 14 days) in the Pilocarpine-induced TLE led to significant suppression of seizure frequency. Moreover, PLX3397 administration (3 or 30 mg/kg per day; for 4 days) reduced the duration of hippocampal paroxysmal discharges (HPDs) in an IHKA model of TLE. Consistently, treatment with PLX3397 (1μM for 14 days) in the organotypic hippocampal slice culture (OHSC) model of epilepsy also reduced epileptiform activity measured by multi-electrode arrays [137]. On the contrary, neither single acute dose PLX3397 treatment nor chronic (for 5 days) pre-treatment with PLX3397 exerts an anti-convulsant effect in any of the acute seizure models, including the maximal electroshock model (MES), Pentylenetetrazol (PTZ), and 6 Hz psychomotor seizure models [137]. This finding reflects that the anti-convulsant effects of PLX3397 are disease context-specific [137].

Along with microglia, a depletion strategy using PLX3397 also reduces astrocyte activation as evidenced by reduced IBA1 and GFAP-positive cells in the PLX3397-treated group, reflecting the potential of PLX3397 to reduce KA-induced astrocyte activation simultaneously with microglia depletion [13].

PLX5622 administration facilitated seizure occurrence and hippocampal damage in the viral encephalitis-induced seizure model, reflecting the protective role of microglia, at least during the initial phase of virus infection [138]. Researchers have hypothesized that increased IL-6 in the brain and IFNγ expression in the spinal cord might contribute to neurodegeneration and underlie the increased seizure susceptibility [138]. Microglia depletion with PLX5622 makes mice more sensitive to CNS infection with Theiler’s murine encephalomyelitis virus (TMEV), leading to the development of seizures and paralysis in PLX5622-treated mice, accompanied by demyelination and axonal damage [139]. Microglia depletion using PLX5622 in a Pilocarpine-induced model of SE reduced astrogliosis and reduced increased seizure susceptibility during the early phase after SE [93]. However, all these studies did not investigate the effect of repopulated microglia on seizures. Nevertheless, these findings suggest the protective and beneficial role of microglia in regulating seizures and epilepsy.

There have been discrepancies in the findings with PLX5622 in terms of the study investigating microglial contribution in epileptogenesis. PLX5622 is a more specific and brain-penetrant CSF-1R inhibitor compared to PLX3397 [140]; however, PLX5622 administration only partially depleted microglia in the unilateral intracortical KA-induced TLE-hippocampal sclerosis (HS) model [50]. More importantly, microglia depletion decreases the severity of KA-induced SE, as evidenced by significantly fewer seizures, reduced time spent in ictal activity, reduced spike number, and high-frequency activity in the PLX5622-treated group. This finding is different from other studies, which have reported that microglia depletion worsens/aggravates seizures [14]. This might be due to the fact that partial microglia depletion by PLX5622 prevents the phenotypic changes in microglia, resulting in a beneficial effect of PLX5622 on KA-induced SE [50]. Moreover, the discrepancy might plausibly be attributed to several factors including the differences in animal models and the efficiency of microglial depletion [50].

Despite the extensive use of PLX3397 and PLX5622 to deplete microglia, emerging evidence reports off-target effects of PLX3397 on oligodendrocyte progenitor cell (OPC) viability [141]. Treatment with high concentrations of PLX3397 and PLX5622 showed cytotoxic effects to OPCs ex vivo and in vitro, as evidenced by a reduction in NG2^+^ or PDGFRα^+^ cells. However, treatment with PLX3397 but not with PLX5622 resulted in a significant reduction in NG2^+^ cells (in cortex, striatum, and spinal cord), reflecting the selective effect of microglia depletion on OPC viability in vivo [141]. PLX5622 has low binding affinity for PDGFRα, which is crucial for OPC development and maintenance [142]; hence, PLX5622 does not interfere with OPC viability at low concentrations (in vitro, ex vivo, or in vivo) [141]. Before precisely assessing the microglial role in epilepsy via depleting microglia with CSF-1R inhibitors, its effect on border-associated macrophages (BAMs), some peripheral immune subsets, its non-CNS effects, and its effect on CNS development and homeostasis should be analyzed [143,144]. This limitation of pharmacological depletion strategies is overcome by genetic depletion strategies, offering higher efficiency and fewer side effects on peripheral immune cells [145].

The difference in the phenotypes of PLX3397 and PLX5622 and genetic depletion used in the above-discussed study is crucial to know. The study reporting the anti-epileptic effect of PLX3397 on acute and chronic seizure models [137] only used one-sixth of the doses needed for microglia depletion [135,146], raising the question of effective microglia depletion and impacting microglia activation and its morphology [137]. However, based on the neuroprotective effect of PLX5622 against seizures [138,139], it can be hypothesized that CSF1R inhibition might have an anti-seizure effect, whereas microglia deletion might be neuroprotective [14]. The activation phenotype of repopulated microglial cells after their depletion, i.e., whether they exist in an activated phenotype or adopt a resting phenotype, possibly promoting the regenerative environment and ameliorating seizure pathogenesis, is also yet to be fully understood [147].

## 5. Dual Effects in Epilepsy: Pro-Epileptic and Anti-Epileptic Effects

**Figure 1:**
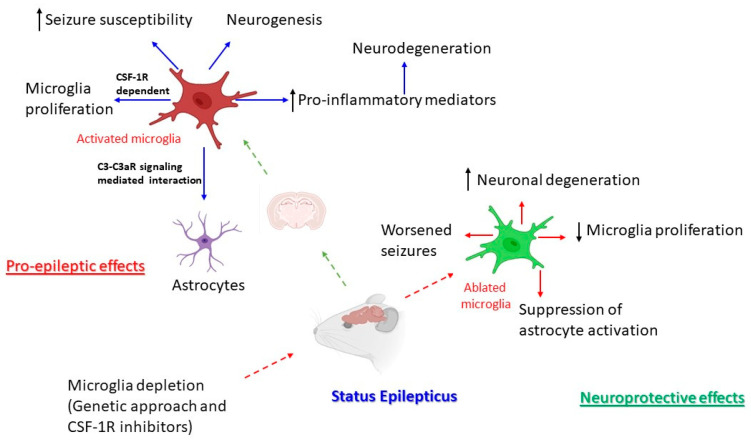
**Microglia dual effects in seizure.** Microglia have gained much attention in pre-clinical epilepsy research due to their pro-epileptic and anti-epileptic/neuroprotective roles. Acquired epilepsy or chemoconvulsive-induced SE leads to the activation of microglia and astrocytes, leading to C3-C3aR signaling-mediated microglia–astrocyte interaction, CSF-1R-dependent microglia proliferation, increased seizure susceptibility, increased neurogenesis, and increased pro-inflammatory mediators leading to neurodegeneration. On the contrary, the beneficial effects of microglia have been outlined upon microglia depletion using pharmacogenetic and pharmacological techniques (CSF-1R inhibitors). This leads to significant ablation of microglia, worsened seizure outcomes, suppression of astrocyte activation, reduced microglial proliferation, and increased neuronal degeneration.

Microglia are acknowledged to contribute to epileptogenesis mainly via their activation and morphological changes (hyper-ramified or ameboid), in turn contributing to neuroinflammation and subsequent neurodegeneration [26,60]. However, microglia can also trigger an epileptic seizure in a manner not dependent on neuroinflammation [28]. In contrast to the current understanding, where microglia have detrimental effects during epilepsy mainly via contributing to chronic neuroinflammation [148], neuroprotective aspects of microglia have been documented as evident by worsened SE-induced seizures upon genetic depletion of microglia [14]. A similar line of effects has also been observed upon pharmacological depletion of microglia using CSF-1R inhibitors (PLX3397, PLX5622, and GW2580) [10,50,93,137]. The pro-epileptic and anti-epileptic phenotypes of microglia (Figure 1) depend on their activation state, meaning that short-term microglial activation is beneficial [130,149] whereas long-term microglial activation is detrimental [150]. It is also crucial to consider that the aspect of microglial activation might be different from the difference in the seizure model induced by different pro-convulsants. Moreover, these dual effects of microglia might be attributed to a direct mechanism in which microglia confer a protective role by maintaining glucose homeostasis and inhibiting ictogenesis, and to an indirect mechanism in which microglia depletion causes nonspecific effects and may plausibly increase seizure susceptibility [147]. One plausible reason might be the dual effects of microglia on BBB permeability, since microglia protect the integrity of the BBB by migrating to the BBB, and later initiate leakage by phagocytosis of BBB components, leading to neuroinflammation [151].

## 6. Therapeutic Targeting of Microglia in Epilepsy

Chronic microglial activation is a hallmark of many CNS disorders, including epilepsy, where the activated microglia contribute to neuroinflammation via releasing pre-inflammatory and neurotoxic mediators, in turn leading to exacerbation of neurotoxicity and neurodegeneration [152]. Several key molecular pathways relevant to the therapeutic targeting of microglia have been identified. These include inflammation inhibiting Minocycline, inhibitors of cytokine pathways and complement cascade, the antagonist of purinergic signaling, and modulators of CX3CL1 signaling, CSF-1R inhibitors, activators of TREM2, etc. [20]. Broadly, microglia depletion, microglia repopulation, and microglia replacement have emerged as therapeutic tools to manipulate microglia [153]. Among these, the widely used strategy to target microglia is to change the microglia landscape with CSF-1R inhibitors via depleting microglia and Minocycline to inhibit microglia activation. More detailed discussion can be found in Section 4 regarding microglia depletion with CSF-1R inhibitors and its effect on seizure outcome. However, repopulated microglia might sometimes be detrimental [154,155], which could be plausibly due to global macrophage depletion by CSF-1R inhibitors. Assessing the short-term and long-term impact of microglia deletion, repopulation, and replacement (with optimal timing) on brain function and cellular interactions can maximize the beneficial effects and minimize the adverse effects [153].

Minocycline has gained more attention as a broad inhibitor of microglia and is a widely used drug in the clinic and has been investigated in the context of epilepsy [156,157]. Minocycline is a semisynthetic tetracycline antibiotic analog and is known for its inhibitory effect on microglial activation, inhibition of microglial phagocytosis of the synapse, and penetration of BBB effectively [22,158]. Minocycline has demonstrated its neuroprotective effects in many neurodegenerative diseases (NDs) including TBI [159], Alzheimer’s disease (AD) [160], Parkinson’s disease (PD) [161], depression [158], etc., mainly via inhibiting microglial activation.

Minocycline has inhibited SE-induced microglial activation without affecting astrocyte activation, inhibited SE-induced hippocampal neuronal loss, and reduced SE-induced activation of the inflammatory mediator (IL-1β and TNF-α) [72]. More importantly, Minocycline treatment reduced the SRS parameter (frequency, duration, and severity), reflecting that Minocycline might exert disease modification against epilepsy [72]. In addition, Minocycline administration has suppressed the long-term anti-epileptogenic effects of early-life seizures mainly by attenuating increased seizure susceptibility and increased activation of microglia [44]. Via its caspase-dependent and independent mechanism, Minocycline treatment has exerted a neuroprotective effect via inhibited KA-induced hippocampal cell death [76]. In the IQSEC3-KD seizure model, Minocycline inhibited DG granule neuron cell death and spontaneous seizures without interfering with GABAergic synapse deficits or somatostatin loss [162]. On a different note, Minocycline treatment does not exert strong anti-convulsive effects in a PTZ kindling model [163], MES [164], and Subcutaneous Metrazol seizure threshold test [164]. No significant protective effect in seizure progression and survival was observed in *Tsc1*^GFAP^CKO mice upon Minocycline treatment despite its potential to inhibit microglial activation in the early stage of diseases [165]. More details on the studies evaluating Minocycline effects against the experimental seizure model are provided in Table 1.

These findings on Minocycline treatment provide compelling evidence that therapeutic treatment targeting activated microglia will not only represent a novel therapeutic strategy to ameliorate neuronal function but also has the potential to halt epileptogenesis via its disease-modifying effect. Before repurposing Minocycline as a future “anti-epileptic therapy” based on its effect from pre-clinical studies, Minocycline is not microglia-specific and having said that, microglia exists in multiple phenotypes, so which phenotypes of microglial activation are inhibited by Minocycline should be fully understood first [167]. The underlying mechanism by which Minocycline acts on microglia rather than on myeloid and non-myeloid peripheral cells remains elusive [167].

Regarding the clinical aspects, several clinical trials targeting microglia with several investigational agents in NDs have emerged rapidly. Clinical trials of several diseases associated with microglial genes, including TREM2, CD33, and *progranulin* (PGRN), via targeting microglia-specific dysfunction have been reported [168]. Several investigational agents of TREM2 (AL002-NCT04592874; VHB937-NCT06643481; VG-3927-NCT06343636; VGL101-NCT05677659), CD33 (AL003-NCT03822208), PGRN (AVB101-NCT06064890; DNL593-NCT05262023), ApoE2 (LX1001-NCT03634007), C1q (ANX005-NCT04569435), CSF-1R (JNJ40346527-NCT04121208), IL-1β (Canakinumab-NCT04795466), NF-kβ (NE3107-NCT04669028), NLRP3 (OLT1177-ISRCTN16806940), TLR2 (NPT52034-NCT03954600), and TNF-α (XPro1595-NCT05318976) have been investigated against several NDs including AD, PD, frontotemporal dementia (FTD), amyotrophic lateral sclerosis (ALS), etc. [168,169,170,171,172,173,174]. Despite promising pre-clinical outcomes of microglia-targeting therapies, translating into clinical settings remains very challenging. This challenge includes less understanding on long-term consequences of microglial depletion, time window, and duration of treatment, along with what stages of disease progression favor microglia transplantation and possible off-target effects [175,176]. Advancement of imaging techniques and development and discovery of biomarker will indeed aid in monitoring microglial dynamics and treatment responses [175].

## 7. Conclusions and Future Implications

The role of microglia in the context of epileptic seizure and its plausible underlying mechanism is complex, warranting future studies. A better understanding of the glial cells, especially microglia, might pave the way forward in delineating the microglial role in seizure generation, which might plausibly represent microglia as a promising therapeutic target against epilepsy. Understanding microglial dynamics, morphologies, and related inflammatory/phagocytic phenotypes during seizure after SE is crucial in developing therapies to manage SE [35]. Many more caveats need to be completely answered before moving to microglia-based therapy as a disease-modifying anti-epileptic strategy. These include the following: (1) A precise understanding of the microglial role in initiating, maintaining, and terminating seizure. (2) Does therapeutically manipulating microglial function prevent seizure occurrence or halt the progression of seizure? (3) What might be the critical window of microglia-based therapeutic intervention against epileptic seizures? [148].

Future microglia-targeting therapies should focus more on reducing the off-target effects, which can be achieved via differentiating individual myeloid populations with the use of advanced tools, and precise understanding of the interaction of different microglial states in health and disease would plausibly drive microglia-targeting therapy [20]. For this, effective microglia-targeting strategies to modulate microglia states (with its receptors, signaling pathways, and channels) that could target activities including phagocytosis, inflammation, process motility, energy metabolism, etc., are needed [20]. Genetic differences among an individual might lead to different microglial responses, and manipulating microglial activity without disrupting microglial normal immune functions remains challenging [177].

## Figures and Tables

**Table 1:** Summary of studies reporting the neuroprotective effect of Minocycline against experimental seizure models.

S.N.	Seizure Model	Minocycline Dose	Observations	References
1	Pilocarpine (40 mg/kg, I.P.) induced SE in SD rats	45 mg/kg, I.P.; for 14 days post-SE	Minocycline treatment inhibited SE-induced microglial activation and SE-induced brain inflammation as evidenced by reduced IBA1-positive microglia and reduced IL-1β and TNF-α respectively in the CA1 and cortex regions without interfering with astrocyte activation. Minocycline treatment prevents SE-induced neuronal loss as evidenced by increased NeuN-positive cells in CA1 and cortex regions. Minocycline treatment attenuates the development of SRS after SE as evidenced by the reduced frequency and duration of SRS and less percentage of stage 5 seizures.	[72]
2	KA (20 mg/kg, I.P.) induced SE in CX3CR1^GFP/+^ mice	20 mg/kg, I.P. 3 h post-KA-SE induction; for 6 consecutive days	Minocycline post-treatment inhibited early-life seizure-induced activation of microglial cells. Treatment with Minocycline prevented the priming effect of an initial seizure and reduced exacerbation of microglia activation, reflecting that Minocycline administration suppressed the increased predisposition to the second seizure later in life.	[44]
3	Intrahippocampal KA (0.4 µL, 1 mg/mL) induced TLE model in male ICR mice.	45 mg/kg, I.P. injected 12 h prior to KA	Minocycline treatment inhibits hippocampal cell death induced by KA as evidenced by increased surviving cells and reduced TUNEL-positive cells (after KA) in the CA1 and CA3 regions in the Minocycline-treated group when compared to vehicle-treated control mice. The underlying mechanism behind this neuroprotection by Minocycline is plausible via inhibition of caspase-dependent and -independent apoptotic pathways.	[76]
4	Amygdala kindling seizure model in male Wistar rats	50, 25, and 12.5 mg/kg, I.P., 60-min before kindling	Minocycline possesses an anti-convulsant effect in fully kindled rats and reduced electrophysiological parameters of after-discharge duration plausibly via inhibiting the neuronal circuits in the amygdala region. Minocycline treatment (50 mg/kg) 1 h after kindling reduced after-discharge duration, the inverse of latency to onset of stage 4, duration of stage 5, and seizure duration. Minocycline (25 mg/kg) treatment reduced after-discharge duration and duration of stage 5, whereas Minocycline (12.5 mg/kg) treatment reduced the duration of stage 5 when compared to the vehicle-treated group. Minocycline treatment (50, 25, and 12.5 mg/kg) 24 h after kindling reduced the duration of stage 5 but did not change the duration of seizure duration and latency to the onset of stage 4.	[166]
5	Animal model of partial, tonic, and tonic–clonic seizure (6 hz test, MES model, and Subcutaneous Metrazol seizure threshold test)	75, 100 and 150 mg/kg, I.P.	Minocycline treatment (75, 100, and 150 mg/kg) was protected from clonic seizures in the 6 hz test, and the effect mainly peaked at 30 min. Minocycline treatment does not exert protection in other seizure models including MES and Subcutaneous Metrazol seizure threshold test.	[164]
6	Mouse model of tuberous–sclerosis complex in *Tsc1*^GFAP^CKO mice	50 mg/kg, I.P./day for 6 days	Treatment with Minocycline inhibited microglial activation in 4-week-old *Tsc1*^GFAP^CKO mice as evidenced by the reduced microglial cell sizes and their number but does not exert any effect on astrocyte proliferation in *Tsc1*^GFAP^CKO mice, reflecting that Minocycline is only selective to microglia. Despite its potential to inhibit microglial activation, Minocycline does not exert any protective effect on seizure progression and survival in *Tsc1*^GFAP^CKO mice.	[165]
7	IQSEC3-KD seizure model	50 mg/kg, I.P.	Minocycline treatment (for 2 weeks) effectively blocked microglial activation as evidenced by reduced IBA1 and CD68-positive cells. Treatment with Minocycline decreased the number and duration of spontaneous ictal seizures and the number of ictal events, reflecting that microglial activation contributes to the occurrence of seizures in IQSEC3-KD mice.	[162]
8	PTZ (37.5 mg/kg, I.P.) induced seizure in NMRI mice	25 mg/kg, I.P.; 1 h. after or before PTZ	Minocycline showed a weak anti-epileptogenic effect via inhibiting the increase in GABAA and NMDA receptor subunits, which is further associated with the reduction in TNF-α receptor expression.	[163]
9	KA (30 mg/kg, I.P.)-induced SE	75 and 50 mg/kg, I.P.; 4 days after SE	Minocycline treatment promoted the survival of the newborn cells mainly via inhibiting microglial activation as evidenced by a reduced number of engulfed newborn cells after SE.	[30]

## Data Availability

No new data were created or analyzed in this study.

## Abbreviations

The following abbreviations are used in this manuscript:

CNS	Central nervous system
SRSs	Spontaneous recurrent seizures;
ASMs	Anti-seizure medications
SE	Status epilepticus
DRE	Drug-resistant epilepsy
MTLE	Mesial temporal lobe epilepsy
BBB	Blood–brain barrier
NDs	Neurodegenerative diseases
SVZ	Subventricular zone
OPCs	Oligodendrocyte progenitor cells
CSF1-R	Colony-stimulating factor 1-receptor
HPDs	Hippocampal paroxysmal discharges
OHSC	Organotypic hippocampal slice culture
KA	Kainic acid
PTZ	Pentylenetetrazol
MES	Maximal electroshock model
ICV	Intracerebroventricular
I.P.	Intraperitoneal
HS	Hippocampal sclerosis
aCSF	Artificial cerebrospinal fluid
DG	Dentate gyrus
ECSs	Electro-convulsive seizures
TLR2	Toll-like receptor 2
MPP	Microglial process pouches
MPC	Microglial process convergence
DREADD	Designer receptor exclusively activated by designer drugs
CCR2	C-C chemokine receptor type 2
PNES	Psychogenic non-epileptic seizures
NORSE	New-onset refractory status epilepticus
CNO	Clozapine N-oxide
